# Overexpression of *BBX18* Promotes Thermomorphogenesis Through the PRR5-PIF4 Pathway

**DOI:** 10.3389/fpls.2021.782352

**Published:** 2021-11-24

**Authors:** Geonhee Hwang, Jeeyoon Park, Soohwan Kim, Jeonghyang Park, Dain Seo, Eunkyoo Oh

**Affiliations:** Department of Life Sciences, Korea University, Seoul, South Korea

**Keywords:** *BBX18*, PRR5, thermomorphogenesis, PIF4, high temperature

## Abstract

Thermomorphogenesis is the morphological response of plants to an elevation in the ambient temperature, which is mediated by the bHLH transcription factor PIF4. The evening-expressed clock component, PRR5, directly represses the expression of *PIF4* mRNA. Additionally, PRR5 interacts with PIF4 protein and represses its transactivation activity, which in turn suppresses the thermoresponsive growth in the evening. Here, we found that the B-box zinc finger protein, BBX18, interacts with PRR5 through the B-Box2 domain. Deletion of the B-Box2 domain abolished the functions of BBX18, including the stimulation of *PIF4* mRNA expression and hypocotyl growth. Overexpression of *BBX18*, and not of *B-Box2-deleted BBX18*, restored the expression of thermoresponsive genes in the evening. We further show that BBX18 prevents PRR5 from inhibiting PIF4-mediated high temperature responses. Taken together, our results suggest that BBX18 regulates thermoresponsive growth through the PRR5-PIF4 pathway.

## Introduction

Plants adapt to high-temperature stresses in various ways, one of which is through morphological changes. The morphological changes in response to elevated ambient temperatures are collectively called thermomorphogenesis (Quint et al., 2016; Casal and Balasubramanian, 2019). Thermomorphogenesis is characterized by elongated hypocotyls, petioles, and primary roots, and leaf hyponasty. These morphological alterations are likely to increase survival during heat stress, partly by enhancing leaf cooling capacity (Crawford et al., 2012). Although thermomorphogenesis is an adaptive response that enables plants to mitigate the damages caused by high temperatures, it is an irreversible and energy intensive process. It is controlled by the integration of multiple environmental and endogenous signals, and ambient temperature.

PHYTOCHROME-INTERACTING FACTOR 4 (PIF4) is a bHLH transcription factor that mediates high temperature-dependent gene expression and alternative splicing events to trigger thermomorphogenesis (Franklin et al., 2011; Sun et al., 2012; Jin et al., 2020). Over the last decade, significant progress has been made in understanding how PIF4 activity and cellular levels are regulated by temperature. The expression of *PIF4* mRNA is elevated in response to high temperatures (Koini et al., 2009; Zhu et al., 2016), a process that is mediated by the evening complex (EC), comprising EARLY FLOWERING 3 (ELF3), ELF4, and LUX ARRHYTHMO (LUX) (Ezer et al., 2017). EC represses the expression of *PIF4* by directly binding to the *PIF4* promoter (Nusinow et al., 2011). High temperatures relieve the EC-mediated *PIF4* repression by inducing ELF3 degradation and attenuating EC binding to the *PIF4* promoter (Ezer et al., 2017; Silva et al., 2020; Zhang et al., 2021). A prion-like domain in ELF3 was proposed to directly mediate temperature-dependent EC binding to target promoters through phase transition (Jung et al., 2020). The protein stability and DNA binding capacity of PIF4 are temperature-sensitive (Kumar et al., 2012; Kim et al., 2020), and are regulated through interactions with the photoreceptor, phytochrome B (phyB) (Lorrain et al., 2008; Park et al., 2018). The phyB activity is dependent on temperature; therefore, it is considered to act as a thermosensor for the regulation of PIF4-mediated thermomorphogenesis (Jung et al., 2016; Legris et al., 2016; Qiu et al., 2019). Other environmental factors (red, blue, and ultraviolet-B light), endogenous hormones (auxin, abscisic acid, brassinosteroid, gibberellic acid, and jasmonic acid), and endogenous energy status affect the activity and/or abundance of PIF4, thereby influencing the thermomorphogenic growth (de Lucas et al., 2008; Oh et al., 2012; Bernardo-Garcia et al., 2014; Ma et al., 2016; Hayes et al., 2017; Ibanez et al., 2018; Martinez et al., 2018; Bellstaedt et al., 2019; Hwang et al., 2019; Xu and Zhu, 2020; Zhu et al., 2021). Although *PIF4* is expressed in all aerial tissues, epidermis is the major tissue that determines the thermomorphogenic growth (Kim et al., 2020). Epidermal PIF4 is activated in response to high temperatures, which in turn induces morphological changes, including hypocotyl elongation by increasing auxin biosynthesis. In addition to PIF4, another phytochrome-interacting bHLH transcription factor, PIF7, activates the thermomorphogenesis pathway (Chung et al., 2020; Fiorucci et al., 2020). High temperatures augment PIF7 protein levels independent of *PIF7* transcription. It was recently revealed that high temperatures enhance the translation of *PIF7* mRNA transcripts through conformational changes in them, which enables rapid accumulation of PIF7 in response to the increased temperatures (Chung et al., 2020).

Thermomorphogenesis is heavily influenced by the circadian clock. The expression of *PIF4* is controlled by the circadian clock (Yamashino et al., 2003; Nozue et al., 2007). The EC, which directly represses *PIF4* expression, mediates the circadian clock as well as the temperature regulation of *PIF4* expression (Nusinow et al., 2011; Ezer et al., 2017). ELF3 directly interacts with PIF4 in an EC-independent manner and prevents PIF4 from activating the expression of its target genes (Nieto et al., 2015). Additionally, two clock components, TIMING OF CAB EXPRESSION 1 (TOC1) and PSEUDO-RESPONSE REGULATOR 5 (PRR5), interact with PIF4 and repress its transactivation activity (Zhu et al., 2016). As a result, high-temperature-mediated PIF4 activation is largely suppressed in the evening when ELF3, TOC1, and PRR5 are expressed at high levels, although the expression of *PIF4* mRNA is highly induced (Zhu et al., 2016). In comparison, PIF4 activates the expression of its target genes in response to increased temperatures at dawn, when the levels of these evening proteins are relatively low (Zhu et al., 2016). The plant-specific protein GIGANTEA (GI) negatively regulates the activity of PIF4 by stabilizing DELLA proteins, which act as negative regulators of PIF4 (de Lucas et al., 2008; Park et al., 2020). Furthermore, GI directly interacts with PIF4, preventing it from binding to the target promoters (Nohales et al., 2019). Given that GI is highly expressed in the evening (Fowler et al., 1999), it is likely that it contributes to the suppression of PIF4-mediated thermomorphogenesis in the evening.

A recent study reported that two B-box zinc finger proteins, B-BOX 18 (BBX18) and BBX23, regulate thermomorphogenesis. The stability of both BBX18 and BBX23 increases at high temperatures (Ding et al., 2018). The expression of *BBX18* is also increased in response to increased temperatures (Ding et al., 2018). Both BBX18 and BBX23 are required for high-temperature-induced hypocotyl growth (Ding et al., 2018). The BBX18 protein directly interacts with ELF3 and negatively regulates its stability, partly through CONSTITUTIVE PHOTOMORPHOGENIC 1 (COP1) (Ding et al., 2018). Given that ELF3 inactivates PIF4 at both the transcriptional and post-translational levels, it is expected that BBX18 promotes hypocotyl growth by potentiating PIF4 activity at high temperatures. However, *PIF4* was not differentially expressed between the wild-type and *bbx18*;*bbx23* double mutants (Ding et al., 2018); therefore, it remains unclear how BBX18 regulates thermomorphogenesis.

Here, we report that BBX18 influences PIF4 to promote hypocotyl growth via ELF3-dependent and -independent pathways. In the ELF3-independent pathway, BBX18 interacts with PRR5 through the B-Box2 domain. This interaction prevents PRR5 from suppressing the PIF4-mediated growth responses, including thermomorphogenesis. Additionally, the expression of *BBX18* is clock-regulated, and peaks at dawn. Our results suggest that an antagonistic interaction between BBX18 and PRR5 regulates PIF4-mediated thermoresponsive growth.

## Materials and Methods

### Plant Materials and Growth Conditions

For general growth and seed harvesting, *Arabidopsis thaliana* plants were grown in a greenhouse with 16 h light/8 h dark cycles at 22°C. All *A. thaliana* plants used in this study were in the Col-0 ecotype background. The full-length coding sequences of *BBX18, BBX18ΔBox1*, and *BBX18ΔBox2* were cloned into the gateway-compatible vector *pX-YFP* (35S promoter-gateway cassette-YFP) to generate transgenic plants *BBX18-OX*, *BBX18ΔBox1-OX*, and *BBX18ΔBox2-OX*. The *bbx18-cr1* and *bbx18-cr2* mutants were generated by transforming the CRISPR-Cas9 construct *pHEE401-UBQ10-BBX18* – targeting the *BBX18* locus – into the wild-type plants (Wang Z. P. et al., 2015). Gene-specific primers used for vector construction are listed in the Supplementary Table 1. The *PRR5-OX* seeds were kindly provided by Nakamichi et al. (2012). The *bbx18-4* (SALK_061956) seeds were kindly provided by Yuan et al. (2021).

### Hypocotyl Length Measurements

Sterilized seeds by 70% (v/v) ethanol and 0.01% (v/v) Triton X-100 were plated on Murashige and Skoog (MS) medium (PhytoTechnology Laboratories) and supplemented with 0.75% phytoagar. After 3 days at 4°C, seeds were treated with white light for 6 h to stimulate germination, and then incubated under specific light and temperature conditions. Seedlings were photographed, and hypocotyl lengths were measured using ImageJ.^1^

### Western Blot Analysis

Plants were harvested and ground in liquid nitrogen. Proteins were extracted using protein extraction buffer (100 mM Tris–HCl of pH 6.8, 25% glycerol, 2% SDS, 0.01% bromophenol blue, and 10% beta-mercaptoethanol). BBX18-YFP protein levels were determined by western blotting using anti-GFP antibody (1:5000 dilution, Takara). PRR5-FLAG protein levels were determined by western blotting using the anti-FLAG antibody (1:2000 dilution, Sigma-Aldrich).

### Co-immunoprecipitation Assays

For co-immunoprecipitation (co-IP) assays using *Arabidopsis* mesophyll protoplasts, 2 × 10^4^ isolated mesophyll protoplasts were transfected with a total of 20 μg of DNA (*35S::PRR5-Myc* and *35S::BBX18-GFP*). The IP buffer was used to lyse the transfected protoplasts (50 mM Tris–Cl pH 7.5, 1 mM EDTA, 75 mM NaCl, 0.1% Triton X-100, 5% glycerol, 1 mM phenylmethylsulfonyl fluoride, 1× protease inhibitor). After centrifugation at 20,000 *g* for 10 min, the supernatant was incubated for 1 h with anti-GFP antibody (A11122, Thermo Fisher Scientific) immobilized on magnetic beads (Dynabeads™ Protein G, Thermo Fisher Scientific). After three washes with IP buffer, the immunoprecipitated proteins were eluted and immunoblotted with anti-Myc (1:5000 dilution, Cell Signaling Technology) and anti-GFP (1:5000 dilution, Takara) antibodies.

### Yeast Two-Hybrid Assays

To identify interactions between BBX18 and PRRs, different fragments of *BBX18* or *PRR* complementary DNA were subcloned into the gateway compatible *pGADT7* or *pGBKT7* vectors (Clontech). The resulting yeast constructs containing various fragments of *BBX18* or *PRRs* cDNA were co-transformed into yeast AH109 cells (Clontech). The yeast clones were grown on synthetic dropout media deficient in Leu and Trp (-LT) and in Leu, Trp, and His (-LTH) but containing various concentrations of 3-amino-1,2,4-Triazole (3-AT).

### Quantitative Real-Time PCR Gene Expression Analysis

Total RNA was extracted from plants using the MiniBEST Plant RNA Extraction Kit (Takara) according to the manufacturer’s instructions. For cDNA synthesis, M-MLV reverse transcriptase (Thermo Fisher Scientific) was utilized. We performed quantitative real-time PCR (qRT-PCR) using the Bio-Rad CFX96 Real-Time PCR detection system and the EvaGreen master mix (Solgent). The expression of each gene was normalized to that of *PP2A*. The gene-specific primers are listed in the Supplementary Table 1.

### Chromatin Immunoprecipitation Assays

The seedlings were cross-linked for 20 min with 1% formaldehyde under vacuum. After cross-linking, seedlings were ground in liquid nitrogen. The cross-linked chromatin complex was resuspended in nuclear lysis buffer (50 mM HEPES at pH 7.5, 150 mM NaCl, 1 mM EDTA, 1% Triton X-100, 0.1% Na deoxycholate, and 0.1% SDS) and sheared by sonication to reduce the average DNA fragment size to approximately 0.3–0.5 kb. The fragmented chromatin complex was then immunoprecipitated using the anti-FLAG antibody (Sigma-Aldrich). The beads were washed with low-salt buffer (50 mM Tris–HCl at pH 8.0, 2 mM EDTA, 150 mM NaCl, and 0.5% Triton X-100), high-salt buffer (50 mM Tris–HCl at pH 8.0, 2 mM EDTA, 500 mM NaCl, and 0.5% Triton X-100), LiCl buffer (10 mM Tris–HCl at pH 8.0, 1 mM EDTA, 0.25 M LiCl, 0.5% NP-40, and 0.5% deoxycholate), and TE buffer (10 mM Tris–HCl at pH 8.0, and 1 mM EDTA), and eluted with the elution buffer (1% SDS and 0.1 M NaHCO_3_). After reverse cross-linking, the eluted DNA fragments were purified using a PCR purification kit (Macherey-Nagel) and quantified by real-time PCR using specific primers (Supplementary Table 1).

## Results

### Overexpression of *BBX18* Promotes Hypocotyl Growth Both at Normal and High Temperatures

**FIGURE 1:**
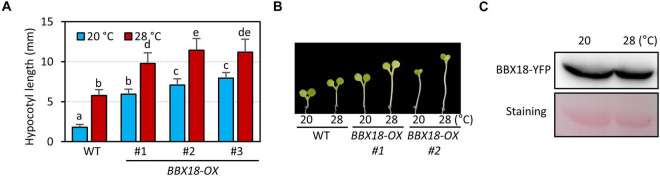
Overexpression of *BBX18* promotes hypocotyl growth at normal and high temperatures. **(A,B)** Hypocotyl lengths of the seedlings [wild-type (WT) and *BBX18-OX* plants] grown under continuous white light at 20°C for 7 days or at 20°C for 4 days followed by 28°C for 3 days. Letters above the bars indicate significant differences based on one-way ANOVA and Tukey’s test (*P* < 0.05). Representative seedlings are shown in **(B)**. **(C)** Levels of BBX18-YFP in *BBX18-OX* plants were not affected by high temperatures. *BBX18-OX* plants were grown under white light constitutively at 20°C or grown at 20°C and shifted to 28°C for 24 h. Immunoblotting was performed using an anti-GFP antibody. Equal loading is demonstrated by Ponceau S staining.

BBX18 was previously shown to promote hypocotyl growth in a temperature-dependent manner (Ding et al., 2018). To confirm this, we generated transgenic plants overexpressing BBX18 fused with YFP at the C-terminus (*BBX18-OX*) and measured the hypocotyl growth of these plants at two temperatures 20 and 28°C. Consistent with the previous report, the hypocotyls of *BBX18-OX* plants were longer than those of the wild-type at 28°C (Figure 1A). However, in contrast to the previous study, we noticed that all three *BBX18-OX* plants had longer hypocotyls than the wild-type at 20°C (Figures 1A,B). Leaf hyponasty was also strongly promoted in the *BBX18-OX* plants at 20°C (Supplementary Figure 1). These results indicate that overexpression of *BBX18* causes constitutive thermomorphogenic responses at normal temperatures. Next, we examined whether BBX18 protein levels are affected by high temperatures. The levels of BBX18-YFP in *BBX18-OX* plants under the two growth temperatures were not significantly different (Figure 1C). These results show that the protein stability of BBX18 is not affected by the ambient temperature changes.

**FIGURE 2:**
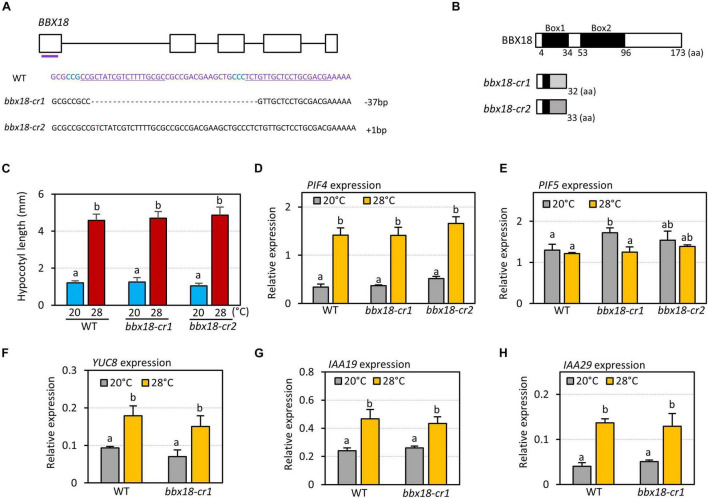
*bbx18* loss-of-function mutants show normal thermoresponsive hypocotyl growth. **(A)** Sequence analysis of CRISPR/Cas9-generated *bbx18* mutations. The gRNA target sequences are depicted by the underlined sequences. The PAM site is in blue. **(B)** Predicted truncated BBX18 proteins in the *bbx18-cr1* and *bbx18-cr2* mutants. aa, amino acids. **(C)** Hypocotyl lengths of WT, *bbx18-cr1*, and *bbx18-cr2* seedlings grown under continuous white light at 20°C for 7 days (20°C) or 20°C for 4 days followed by 28°C for 3 days (28°C). Letters above the bars indicate significant differences based on a one-way ANOVA and Tukey’s test (*P* < 0.05). qRT–PCR analyses to check the expression of *PIF4* **(D)**, *PIF5* **(E)**, *YUC8* **(F)**, *IAA19* **(G)**, and *IAA29* **(H)**. WT and *bbx18* mutants were grown in 12 h light/12 h dark cycles (12L:12D) at 20°C for 4 days. On the 5th day, the plants were shifted to continuous white light and exposed to a high temperature of 28°C for 4 h at ZT20–24 or grown at 20°C before harvesting for RNA extraction. The expression of target genes was normalized to that of *PP2A*. Error bars indicate SD (*n* = 3). Letters above the bars indicate significant differences based on a one-way ANOVA and Tukey’s test (*P* < 0.05).

It was previously shown that hypocotyl growth is hyposensitive to high temperatures in the *bbx18* loss-of-function mutants (Ding et al., 2018). To confirm this result, we generated two *bbx18* loss-of-function mutants (*bbx18-cr1* and *bbx18-cr2*) using CRISPR/Cas9 against *BBX18*. *bbx18-cr1* mutants contained a 37-nucleotide deletion in the first exon, and *bbx18-cr2* mutants had a single-nucleotide insertion in the first exon (Figure 2A). These indel mutations lead to a frameshift and resulted in the generation of premature stop codons (Figure 2B); therefore, both *bbx18-cr1* and *bbx18-cr2* mutants are likely to be null mutants. However, the hypocotyl growth response to high temperatures was not significantly affected in both *bbx18-cr1* and *bbx18-cr2* mutants (Figure 2C). We also examined the thermoresponsive hypocotyl growth of *bbx18-4* mutants with a T-DNA insertion in the 3′-UTR of the *BBX18* gene. The hypocotyl growth in *bbx18-4* mutants at high temperature was comparable to that of the wild-type at both the temperatures (Supplementary Figure 2). In line with these results, the expression of *PIF4* and *PIF5* was not significantly altered in response to the introduction of *bbx18* mutations (Figures 2D,E). Expression of PIF4 target genes (*YUC8*, *IAA19*, and *IAA29*) was similarly induced by high temperature in wild-type and *bbx18-cr1* plants (Figures 2F–H). Together, these results suggest that genes with functions similar to those of *BBX18* might be present in the *Arabidopsis* genome.

### PIFs Are Required for BBX18-Mediated Hypocotyl Growth Promotion

**FIGURE 3:**
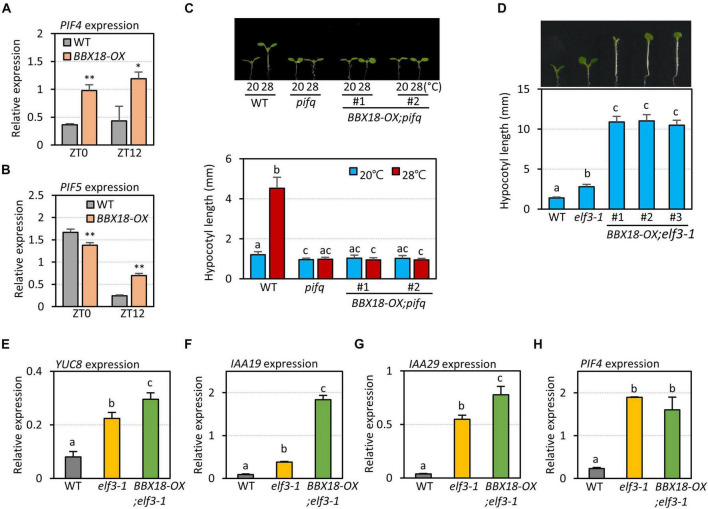
BBX18-mediated promotion of hypocotyl growth is dependent on PIFs, but not on ELF3. qRT–PCR analyses to check for the expression of *PIF4* **(A)** and *PIF5* **(B)**. Wild-type (WT) and *BBX18-OX* plants were grown in 12 h light/12 h dark cycles (12L:12D) at 20°C for 4 days. On the 5th day, the plants were harvested at ZT0 and ZT12 for RNA extraction. The expression of target genes was normalized to that of *PP2A*. Error bars indicate SD (*n* = 3). *P < 0.05 and **P < 0.01 (Student’s *t*-test). **(C)** Hypocotyl lengths of WT, *pifq*, and *BBX18-OX;pifq* seedlings grown under continuous white light at 20°C for 7 days (20°C) or 20°C for 4 days followed by 28°C for 3 days (28°C). Letters above the bars indicate significant differences based on one-way ANOVA and Tukey’s test (*P* < 0.05). **(D)** Hypocotyl lengths of WT, *elf3-1*, and *BBX18-OX;elf3-1* seedlings grown under continuous white light at 20°C for 7 days. Letters above the bars indicate significant differences based on one-way ANOVA and Tukey’s test (*P* < 0.05). qRT–PCR analyses to check for the expression of *YUC8* **(E)**, *IAA19* **(F)**, *IAA29* **(G)**, and *PIF4* **(H)**. WT, *elf3-1*, and *BBX18-OX;elf3-1* #1 plants were grown in 12 h light/12 h dark cycles (12L:12D) at 20°C for 4 days. On the 5th day, the plants were harvested at ZT0 for RNA extraction. The expression of target genes was normalized to that of *PP2A*. Error bars indicate SD (*n* = 3). Letters above the bars indicate significant differences based on one-way ANOVA and Tukey’s test (*P* < 0.05).

A previous study proposed that BBX18 promotes hypocotyl growth by reducing the levels of ELF3, which negatively regulates the expression of *PIF4* and its homolog *PIF5* (Ding et al., 2018). However, it was not shown whether BBX18 influences the expression of these *PIFs* (Ding et al., 2018). Therefore, we determined the expression of *PIF4* and *PIF5* in the wild-type and *BBX18-OX* plants at two zeitgeber times (ZT0 and ZT12). *PIF4* expression was higher in *BBX18-OX* plants than in the wild-type plants at ZT0 and ZT12 (Figure 3A). *PIF5* expression was similar in the wild-type and *BBX18-OX* at ZT0, but was higher in the *BBX18-OX* than in the wild-type at ZT12 (Figure 3B). Given that ELF3 represses the expression of *PIF4* and *PIF5* in the evening, these results corroborate the hypothesis that BBX18-mediated ELF3 degradation stimulates hypocotyl growth through PIF4 and PIF5. To further confirm this hypothesis, we examined if PIFs are necessary for the promotion of hypocotyl growth by BBX18. Overexpression of *BBX18* was not able to promote hypocotyl growth in the *pif* quadruple (*pifq*) mutants lacking PIF1, PIF3, PIF4, and PIF5 at normal temperature (20°C) (Figure 3C). BBX18 could not promote hypocotyl growth even at high temperature (28°C) in the *pifq* mutants (Figure 3C). These results indicate that the promotion of hypocotyl growth by BBX18 is dependent on the PIF activity, irrespective of the temperature.

### BBX18 Interacts With PRR Members

Next, to examine whether BBX18 regulates hypocotyl growth exclusively through the ELF3-dependent pathway, we determined the effects of *BBX18* overexpression on hypocotyl growth in an *elf3-1* mutant background (*BBX18-OX;elf3-1*). Interestingly, overexpressed BBX18 significantly promoted hypocotyl growth in the absence of functional ELF3 (Figure 3D). This finding indicates that BBX18 promotes hypocotyl growth via ELF3-dependent and -independent pathways.

**FIGURE 4:**
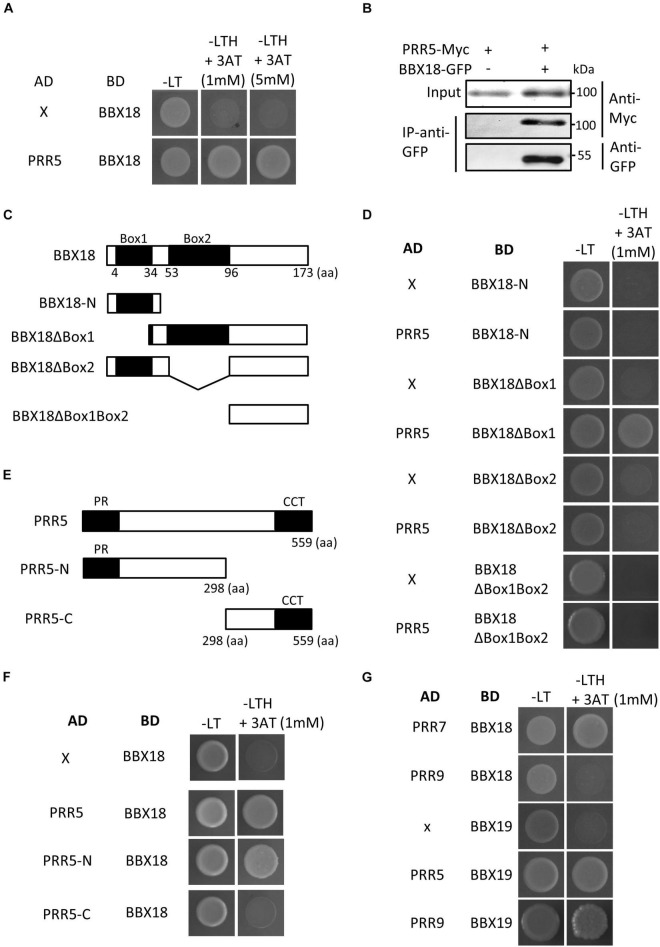
BBX18 interacts with PRR proteins, including PRR5. **(A,D,F,G)** Yeast two-hybrid assays showing the BBXs interacting with PRRs. Yeast clones were grown on synthetic dropout media lacking Leu and Trp (-LT) and lacking Leu, Trp, and His (-LTH), but containing various concentrations of 3-amino-1,2,4-Triazole (3-AT). **(B)** BBX18 interacted with PRR5 *in planta*. Protein extracts from protoplasts expressing PRR5-Myc and PRR5-Myc and BBX18-GFP were immunoprecipitated using the anti-GFP antibody and analyzed by immunoblotting with the anti-GFP and anti-Myc antibody, respectively. The molecular weight (kDa) is indicated on the right side of the gel. **(C)** Box diagram depicting various BBX18 fragments used in the yeast two-hybrid assays in **(D)**. **(E)** Box diagram depicting various PRR5 fragments used in the yeast two-hybrid assays in **(F)**.

Given the inability of BBX18 to enhance hypocotyl growth in the *pifq* mutants (Figure 3C), it is likely that the ELF3-independent pathway also increases PIF abundance and/or activity to induce hypocotyl growth promotion. In line with this hypothesis, the expression levels of PIF4 target genes (*YUC8*, *IAA19*, and *IAA29*) were significantly higher in *BBX18-OX;elf3-1* plants than those in *elf3-1* plants (Figures 3E–G). In contrast, *PIF4* expression was not significantly increased in response to the overexpression of *BBX18* in the *elf3-1* mutant, implying that BBX18 potentiates PIF4 activity (Figure 3H). To identify the molecular mechanism by which BBX18 promotes hypocotyl growth independent of ELF3, we tested the interaction of BBX18 with proteins known to regulate PIF activities using yeast two-hybrid assays (Supplementary Figures 3A,B). This screening identified PRR5 as a BBX18-interacting protein (Figure 4A). PRR5 was previously shown to inhibit the transactivation activity and transcript-level expression of PIF4 (Nakamichi et al., 2012; Zhu et al., 2016). We confirmed the interaction between BBX18 and PRR5 *in vivo* using co-IP assays (Figure 4B). BBX18 contains two tandem repeats of B-Box domains (B-Box1 and B-Box2). To identify the domains of BBX18 which are required for interaction with PRR5, we performed the yeast two-hybrid assays with the truncated versions of BBX18 proteins (Figures 4C,D). The assays revealed that the B-Box2 domain is necessary for the interaction of BBX18 with PRR5 (Figure 4D). PRR5 has a pseudo-receiver (PR) domain at the N-terminus and CONSTANTS, CONSTANS-LIKE, and TOC1 (CCT) domains at the C-terminus (Figure 4E). Yeast two-hybrid assays revealed that the PRR5 truncation containing the PR domain, and not the CCT domain, interacted with BBX18 (Figure 4F).

We next examined whether PRR5 interacts with BBX19, a homolog of BBX18, which promotes hypocotyl growth through PIFs (Wang C. Q. et al., 2015). Figure 4G shows that PRR5 interacts with BBX19 as well as BBX18. Additional yeast two-hybrid assays revealed that BBX18 interacts with PRR7 but not PRR9, and BBX19 interacts with PRR9. Taken together, these results indicate that BBX18 and BBX19 interact with multiple PRR proteins.

### B-Box2 Domain Is Essential for the Hypocotyl Growth Promoting Activity of BBX18

**FIGURE 5:**
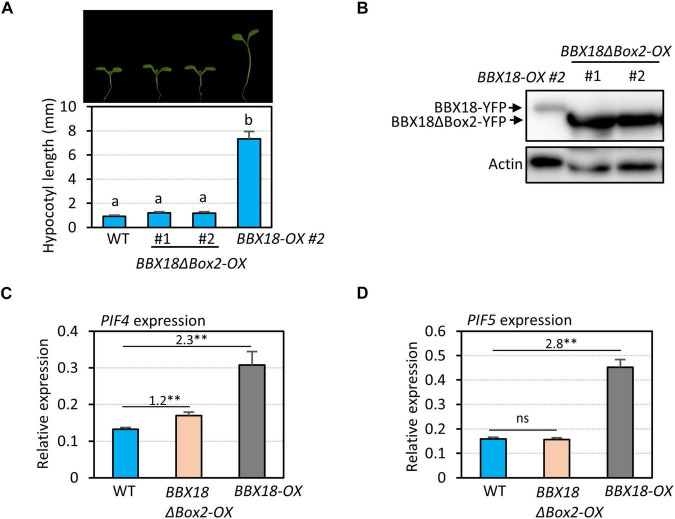
B-Box2 domain is required for the hypocotyl growth promoting activity of BBX18. **(A)** Hypocotyl lengths of WT, *BBX18ΔBox2-OX*, and *BBX18-OX* seedlings grown under continuous white light at 20°C for 7 days. Letters above the bars indicate significant differences based on one-way ANOVA and Tukey’s test (*P* < 0.05). Representative seedlings are shown in the upper panel. **(B)** Immunoblot analyses of BBX18 and BBX18ΔBox2 proteins. The plants were grown under white light constitutively at 20°C and harvested for total protein extraction. Immunoblotting was carried out using an anti-GFP antibody. Equal loading is demonstrated by Ponceau S staining. qRT–PCR analyses to check the expression of *PIF4* **(C)** and *PIF5* **(D)**. WT, *BBX18ΔBox2-OX*, and *BBX18-OX* plants were grown in 12 h light/12 h dark cycles (12L:12D) at 20°C for 4 days. On the 5th day, the plants were harvested at ZT12 for RNA extraction. The expression of target genes was normalized to that of *PP2A*. Error bars indicate SD (*n* = 3). **P < 0.01 (Student’s *t*-test). ns, not significant (Student’s *t*-test *P* ≥ 0.05). Numbers indicate the ratio of gene expression.

In contrast to BBX18, PRR5 inhibits hypocotyl growth by inhibiting the expression of *PIF4* and repressing the transactivation activity of PIF4 (Nakamichi et al., 2012; Zhu et al., 2016). Therefore, it is likely that the BBX18-PRR5 interaction interferes with PRR5 to promote hypocotyl growth. To test this hypothesis, we first determined whether the B-Box2 domain is required by BBX18 to promote hypocotyl growth because the B-Box2 domain mediates the BBX18-PRR5 interaction. We generated transgenic plants overexpressing BBX18 with the deletion of B-Box2 (*BBX18ΔBox2-OX*). In contrast to *BBX18-OX* plants, the hypocotyls of *BBX18ΔBox2-OX* plants were not significantly longer than those of wild-type plants (Figure 5A), although the levels of BBX18ΔBox2-YFP were higher than those of BBX18-YFP (Figure 5B). Consistent with this hypocotyl growth pattern, the overexpression of *BBX18ΔBox2* only marginally increased *PIF4* expression and had no effect on the expression of *PIF5* (Figures 5C,D). Given that the B-Box2 domain is required for the interaction of BBX18 with PRR5 (Figure 4D), BBX18 may promote hypocotyl growth, at least partially, through the inhibition of PRR5. Additionally, we generated transgenic plants overexpressing BBX18 with the deletion of the B-Box1 domain (*BBX18ΔBox1-OX*) and measured their hypocotyl growth. The overexpression of *BBX18ΔBox1* did not affect hypocotyl growth (Supplementary Figure 4). The B-Box1 domain of BBX19 was previously shown to be essential for its interaction with ELF3 (Wang C. Q. et al., 2015). Therefore, the deletion of the B-Box1 domain is likely to abolish the interaction between BBX18 and ELF3, which might be partially responsible for the inability of BBX18ΔBox1 to promote hypocotyl growth.

### BBX18 Does Not Interfere With the Binding of PRR5 to the *PIF4* Promoter

PRR5 directly binds to the promoter of *PIF4* and represses its expression (Nakamichi et al., 2012). Because BBX18 augments *PIF4* expression (Figure 3A), we examined whether BBX18 promotes *PIF4* expression by preventing PRR5 from binding to the *PIF4* promoter using chromatin immunoprecipitation (ChIP) assays in *PRR5-OX* and *PRR5-OX;BBX18-OX* plants. Consistent with the findings of a previous report (Nakamichi et al., 2012), ChIP assays showed that PRR5 binds to the *PIF4* promoter (Supplementary Figure 5). The binding of PRR5 to the *PIF4* promoter was not significantly affected by the overexpression of *BBX18* (Supplementary Figure 5). These results show that the interaction between BBX18 and PRR5 does not affect the DNA-binding ability of PRR5.

### BBX18 Prevents PRR5 From Inhibiting Thermomorphogenesis

**FIGURE 6:**
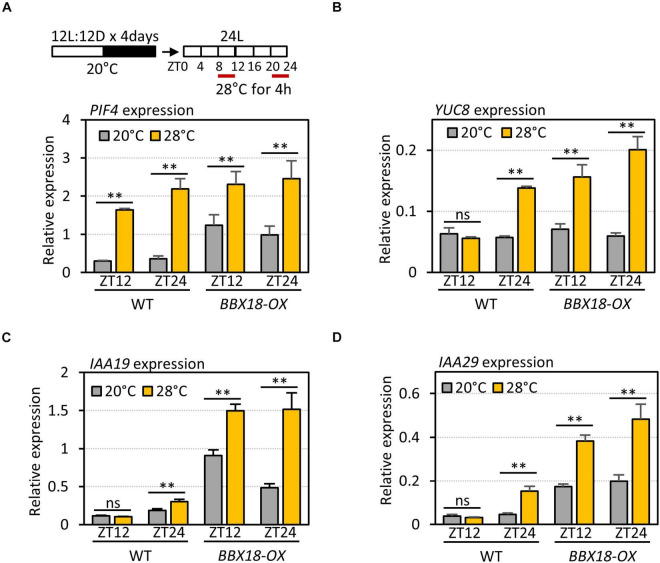
Overexpression of *BBX18* restores thermosensitivity in the evening. qRT–PCR analysis to check the expression of *PIF4* **(A)**, *YUC8* **(B)**, *IAA19* **(C)**, and *IAA29* **(D)**. WT and *BBX18-OX* plants were grown in 12 h light/12 h dark cycles (12L:12D) at 20°C for 4 days. On the 5th day, the plants were shifted to continuous white light and exposed to a high temperature (28°C) for 4 h at different ZTs (ZT8–12 and ZT20–24) before harvesting for RNA extraction. The expression of target genes was normalized to that of *PP2A*. Error bars indicate SD (*n* = 3). *P < 0.05 and ^**^P < 0.01 (Student’s *t*-test). ns, not significant (Student’s *t*-test *P* ≥ 0.05).

The expression of *PRR5* is regulated by the circadian clock, and peaks at ZT12. The circadian clock-dependent accumulation of PRR5 mediates the circadian gating of high-temperature-mediated PIF4 activation and thermoresponsive hypocotyl growth (Zhu et al., 2016). High-temperature-mediated PIF4 activation is enhanced around ZT0 when the PRR5 levels are low, whereas it is suppressed around ZT12 when the PRR5 levels are high (Zhu et al., 2016). To test whether the interaction between BBX18 and PRR5 alleviates PRR5-mediated suppression of high-temperature responses, we first determined the high-temperature responses of *PIF4* and PIF4 target genes – in terms of expression – at different circadian times (ZT8–12 and ZT20–24) in wild-type and *BBX18-OX* plants. Although the expression of *PIF4* was similarly induced by high-temperature during ZT8–12 and ZT20–24 (Figure 6A), the expression of several PIF4 target genes (*YUC8*, *IAA19*, and *IAA29*) was increased in response to the high temperature at ZT20–24, but not at ZT8–12, in the wild-type (Figures 6B–D). In contrast, the expression of these PIF4 target genes was induced by high temperature both during ZT8–12 and ZT20–24 in *BBX18-OX* plants (Figures 6B–D). These results show that overexpressed BBX18 restores the thermoresponsiveness of the PIF4 target genes in the evening (ZT12).

**FIGURE 7:**
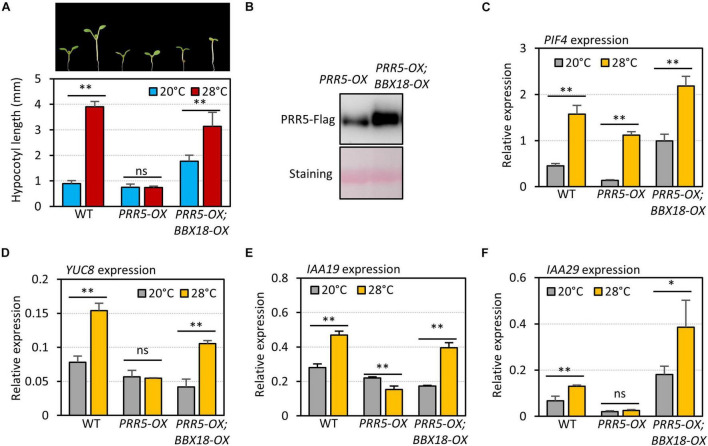
BBX18 prevents PRR5 from inhibiting thermomorphogenesis. **(A)** Hypocotyl lengths of WT, *PRR5-OX*, and *PRR5-OX;BBX18-OX* seedlings grown under continuous white light at 20°C for 7 days (20°C) or 20°C for 4 days followed by 28°C for 3 days (28°C). Error bars indicate SD (*n* = 10 plants). **P < 0.01 (Student’s *t*-test). ns, not significant (Student’s *t*-test *P* ≥ 0.05). Representative seedlings are shown in the upper panel. **(B)** Immunoblot analysis of PRR5-Flag proteins in *PRR5-OX* and *PRR5-OX;BBX18-OX* plants. The plants were grown under continuous white light at 20°C for 4 days followed by 28°C for 3 days and harvested for total protein extraction. Immunoblotting was carried out using an anti-FLAG antibody. Equal loading is demonstrated by Ponceau S staining. qRT–PCR analysis to check for the expression of *PIF4* **(C)**, *YUC8* **(D)**, *IAA19* **(E)**, and *IAA29* **(F)**. WT, *PRR5-OX*, and *PRR5-OX;BBX18-OX* plants were grown in 12 h light/12 h dark cycles (12L:12D) at 20°C for 4 days. On the 5th day, the plants were shifted to continuous white light and exposed to a high temperature (28°C) for 4 h at ZT20–24 or grown at 20°C before harvesting for RNA extraction. The expression of target genes was normalized to that of *PP2A*. Error bars indicate SD (*n* = 3). *P < 0.05 and ^**^P < 0.01 (Student’s *t*-test). ns, not significant (Student’s *t*-test *P* ≥ 0.05).

To examine if BBX18 interferes with PRR5-mediated inhibition of the high-temperature responses, we measured the hypocotyl growth of wild-type, *PRR5-OX*, and *PRR5-OX;BBX18-OX* plants grown under two different temperature conditions (20 and 28°C). As previously reported (Zhu et al., 2016), while the hypocotyls of the wild-type were significantly elongated at high temperature, those of *PRR5-OX* were mostly insensitive to the high temperatures (Figure 7A). The impaired thermoresponsive hypocotyl growth in *PRR5-OX* plants was partially restored in response to the overexpression of *BBX18* (Figure 7A). The levels of PRR5 were not reduced but rather increased upon the overexpression of *BBX18* (Figure 7B), which indicates that the restored thermoresponsive hypocotyl growth in *PRR5-OX*;*BBX18-OX* plants could not be attributed to the reduced levels of PRR5. The expression of PIF4 target genes (*YUC8*, *IAA19*, and *IAA29*) in the *PRR5-OX* plants did not increase at high temperatures, although the expression of *PIF4* was significantly increased (Figures 7C–F), confirming that PRR5 inhibits PIF4-mediated activation of *YUC8, IAA19*, and *IAA29*. The thermoresponsive expression of these genes was restored in *PRR5-OX*;*BBX18-OX* plants (Figures 7D–F), consistent with the hypocotyl phenotypes. Therefore, these results provide evidence that BBX18 inhibits PRR5-mediated suppression of PIF4 activity, concomitantly enhancing the thermoresponsive hypocotyl growth.

## Discussion

BBX18 was previously shown to mediate high-temperature-induced hypocotyl growth by promoting the degradation of ELF3 (Ding et al., 2018). In this study, we found that BBX18 promotes hypocotyl growth in the absence of ELF3, which implies that BBX18 influences the ELF3-independent pathways to enhance the hypocotyl growth. We also found that BBX18 directly interacts with PRR members, including PRR5, which represses both PIF4 transcription activation activity and *PIF4* mRNA expression. The B-Box2 domain in BBX18 was essential for BBX18 to interact with PRR5 and promote hypocotyl growth. Additionally, overexpression of *BBX18* in *PRR5-OX* plants restored the PIF4-mediated thermoresponsive hypocotyl growth and gene expression. Therefore, our work suggests that BBX18 potentiates high-temperature-induced hypocotyl growth by preventing PRR5 from inhibiting PIF4 as well as by inducing the degradation of ELF3.

ELF3 is a component of EC that binds to the promoters of *PIF4* and *PIF5*, and represses the expression of these genes (Nusinow et al., 2011). However, it has not been shown that the interaction of BBX18 with ELF3 influences the expression of *PIFs* (Ding et al., 2018). We found that the expression of both *PIF4* and *PIF5* was increased in *BBX18-OX* plants (Figures 3A,B). Together with the observation that BBX18 could not promote hypocotyl growth in the absence of PIFs (Figure 3C), these findings support the hypothesis that BBX18 enhances thermomorphogenesis by derepressing *PIF4* expression (Ding et al., 2018). Overexpression of *BBX18* failed to activate *PIF4* expression in the *elf3* mutant (Figure 3H), indicating that ELF3 is required by BBX18 to regulate the expression of *PIF4*. In contrast, hypocotyl growth and the expression of PIF4 target genes were increased upon the overexpression of *BBX18* even in the absence of ELF3 (Figures 3D–G), providing evidence that BBX18 potentiates PIF4 activity independent of ELF3.

It has been reported that BBX18 promotes hypocotyl growth only at high temperature (29°C) (Ding et al., 2018). However, we observed that BBX18 significantly promoted hypocotyl growth at normal temperatures (20°C) (Figures 1A,B). The differences in the levels of *BBX18* overexpression in the transgenic plants or growth conditions were likely responsible for these contradictory results. For hypocotyl growth assays, we grew the plants on a medium without sucrose, whereas sucrose-containing medium was employed in a previous study (Ding et al., 2018). However, we observed that *BBX18-OX* plants had longer hypocotyls than the wild-type plants, even when grown on sucrose-containing medium (Supplementary Figure 6A). ELF3 and PRR5 have been shown to affect hypocotyl growth at normal temperatures (Reed et al., 2000; Nakamichi et al., 2005). Additionally, the closest homolog of BBX18, BBX19, has been previously shown to promote hypocotyl growth at 22°C (Wang C. Q. et al., 2015). These results support the ability of BBX18 to promote hypocotyl growth at normal temperatures.

PRR5 is an evening-expressed clock-regulated protein (Nakamichi et al., 2005). In the evening, PRR5 directly binds to PIF4 and represses its transcriptional activity, thereby suppressing PIF4-mediated gene activation and thermoresponsive hypocotyl growth (Zhu et al., 2016). Overexpression of *BBX18* restored the expression of thermosensitive genes in the evening in the wild-type (Figure 6). In addition, it restored the thermo-insensitive growth of the *PRR5-OX* plants (Figure 7). These results indicate that the direct interaction of BBX18 with PRR5 inhibits PRR5 expression and/or activity. As BBX18-OX did not reduce the PRR5 levels, BBX18 is likely to interfere with PRR5 activity by suppressing the transcription repression activity of PRR5 or disrupting the interaction between PRR5 and PIF4. It is therefore worth investigating the molecular mechanisms through which the BBX18-PRR5 interaction derepresses PIF4 activity.

While we were preparing this manuscript, Yuan et al. (2021) reported that BBX18 and BBX19 interact with PRR proteins, which is consistent with our results. They showed that the interaction of BBX19 with the PRRs enables BBX19 to bind to the promoters of *CCA1* and *RVE8* and inhibit the expression of these genes, thereby fine-tuning the circadian rhythm (Yuan et al., 2021). It is possible that the interactions between BBXs and PRRs are involved in other PRR-mediated responses, such as resistance to abiotic stress (Liu et al., 2013; Nakamichi et al., 2016), in addition to the regulation of the circadian clock and thermoresponsive growth.

In addition to *PRR5*, *BBX18*, and *BBX19* are also clock-regulated, and their expression peaks at dawn (Nakamichi et al., 2005). We confirmed the clock-dependent expression of these genes (Supplementary Figure 7). The antiphase in the expression of *PRR5* and *BBX18* might reinforce the circadian clock to gate the thermoresponsive growth by eliminating the remaining activity of PRR5, which ensures that the high-temperature-activated PIF4 promotes hypocotyl growth at dawn. However, thermoresponsive growth and gene expression were not substantially impaired in the *bbx18* loss-of-function mutants (*bbx18-cr1*, *bbx18-cr2*, and *bbx18-4*) (Figure 2 and Supplementary Figure 6B). Other B-Box2 containing BBX proteins, including BBX19, BBX24, and BBX25, are likely to compensate for the absence of BBX18. Thus, further experiments with double or higher-order mutants are required to ascertain the extent to which BBX proteins contribute to the thermoresponsive growth.

## Data Availability Statement

The original contributions presented in the study are included in the article/Supplementary Material, further inquiries can be directed to the corresponding author.

## Author Contributions

GH, JyP, and EO conceived and designed the research, performed the experiments, and wrote the manuscript. SK, JhP, and DS performed the experiments and analyzed the data. All authors contributed to the article and approved the submitted version.

## Conflict of Interest

The authors declare that the research was conducted in the absence of any commercial or financial relationships that could be construed as a potential conflict of interest.

## Publisher’s Note

All claims expressed in this article are solely those of the authors and do not necessarily represent those of their affiliated organizations, or those of the publisher, the editors and the reviewers. Any product that may be evaluated in this article, or claim that may be made by its manufacturer, is not guaranteed or endorsed by the publisher.

## Footnotes

1
http://rsb.info.nih.gov/ij
