# Supplementary figures and images for: Overexpression of BBX18 Promotes Thermomorphogenesis Through the PRR5-PIF4 Pathway
Source: Front Plant Sci. 2021 Nov 24;12:782352. doi: 10.3389/fpls.2021.782352 (PMC8651621; doi:10.3389/fpls.2021.782352)

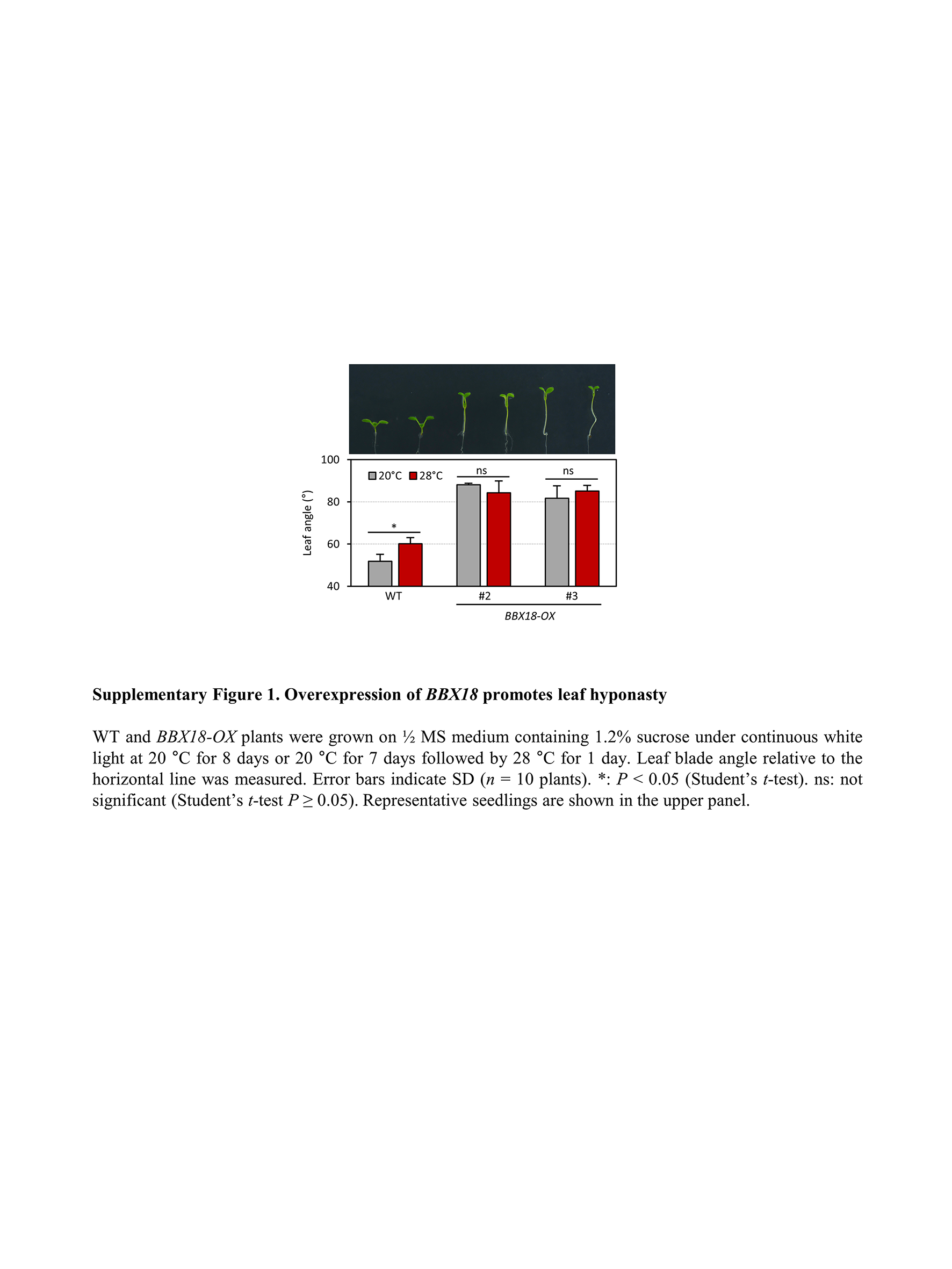

Supplement: Supplementary file 2 [file Image_1.TIF]

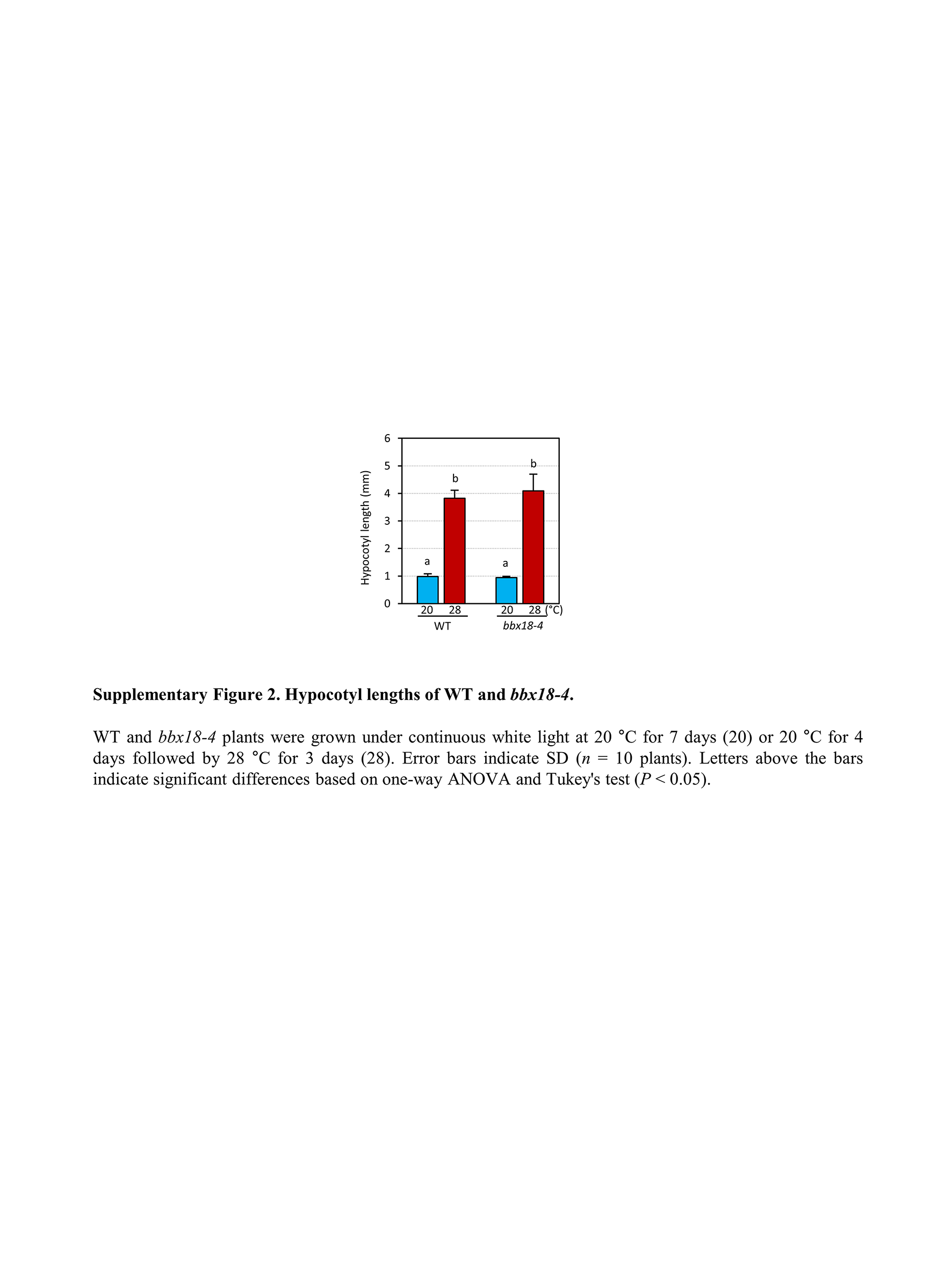

Supplement: Supplementary file 3 [file Image_2.TIF]

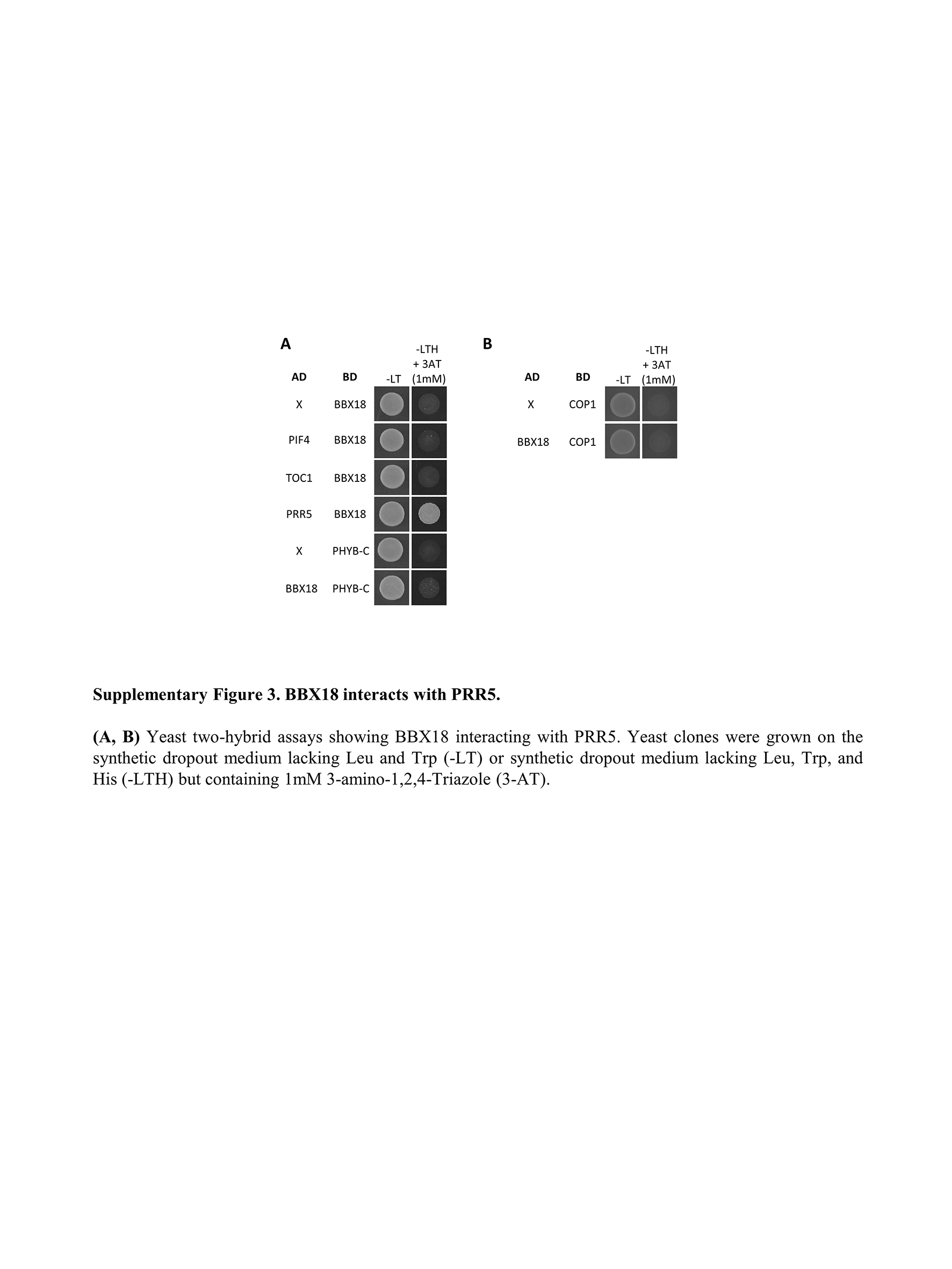

Supplement: Supplementary file 4 [file Image_3.tif]

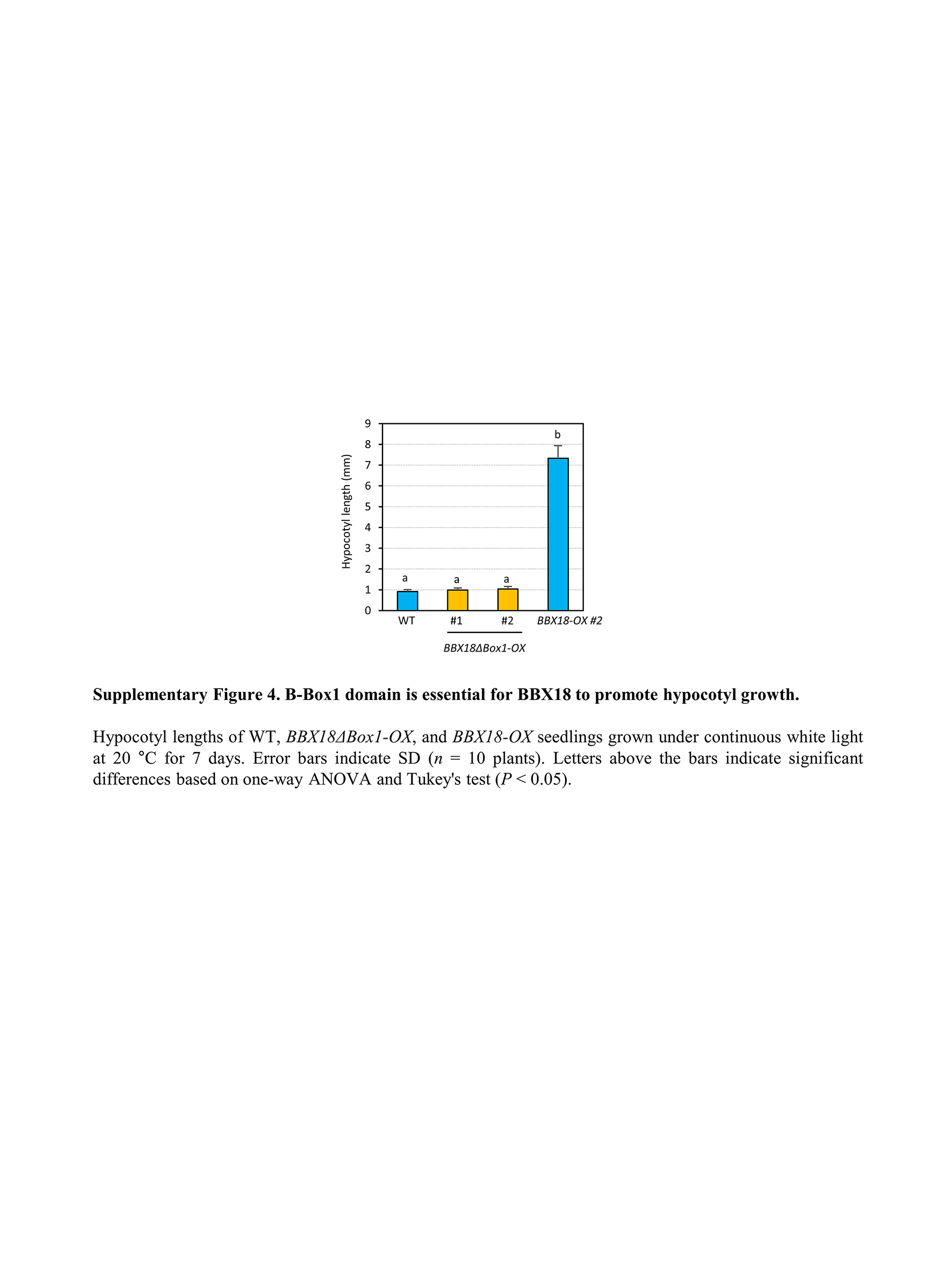

Supplement: Supplementary file 5 [file Image_4.TIF]

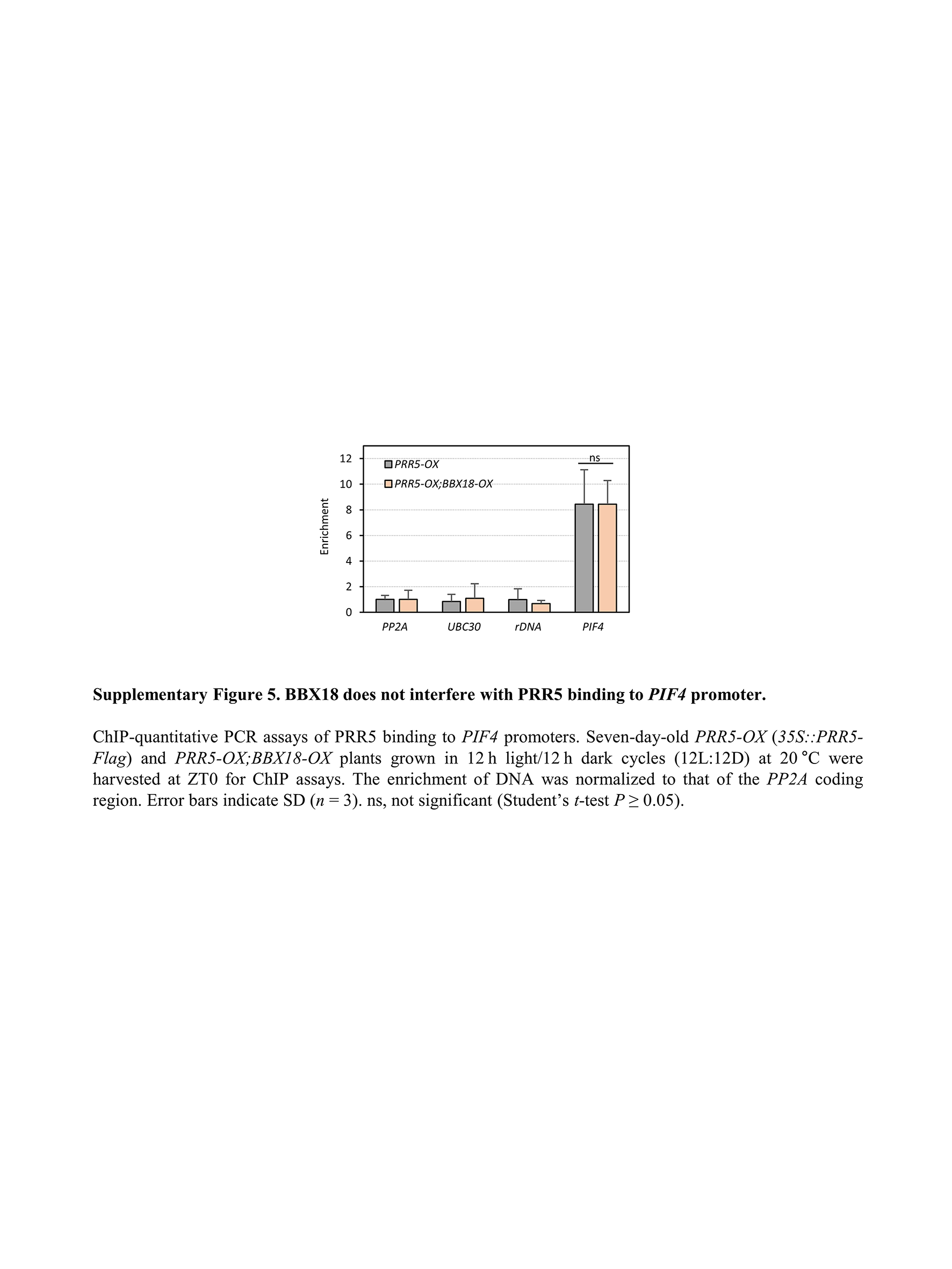

Supplement: Supplementary file 6 [file Image_5.TIF]

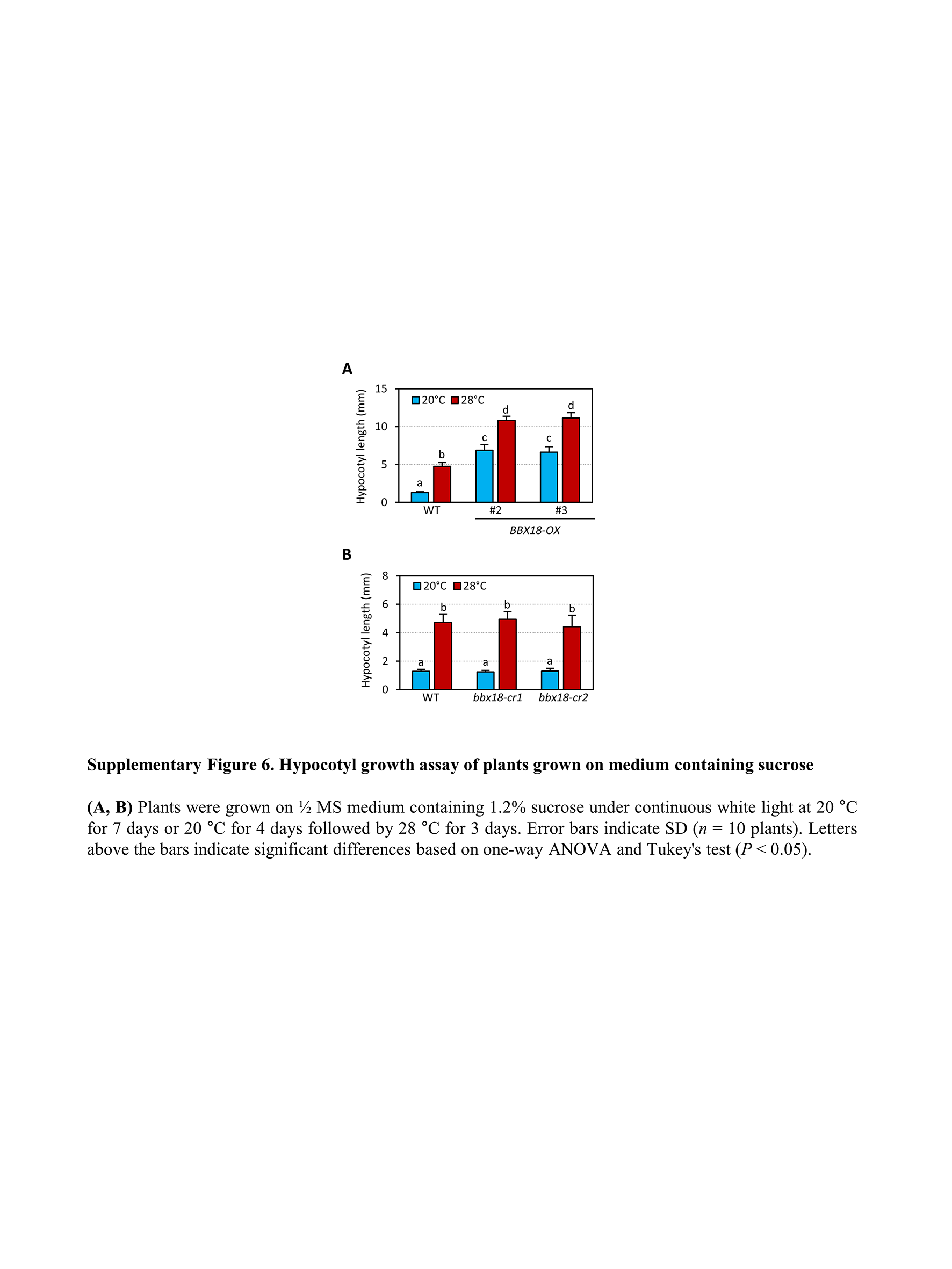

Supplement: Supplementary file 7 [file Image_6.TIF]

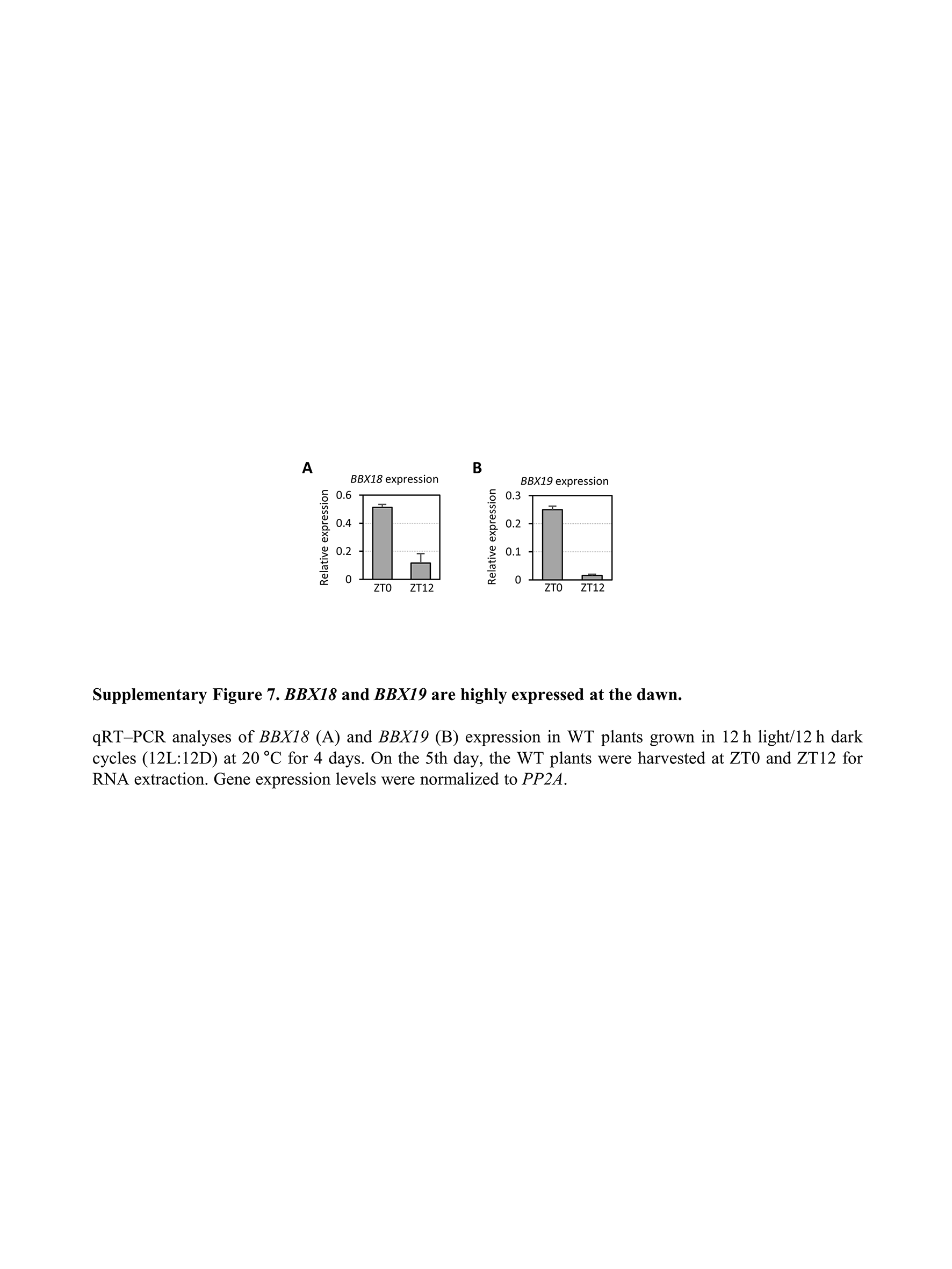

Supplement: Supplementary file 8 [file Image_7.TIF]
